# Understanding the Termination of Urologic Cancer Clinical Trials: Insights and Challenges

**DOI:** 10.1200/GO.23.00349

**Published:** 2024-01-11

**Authors:** Abdulrahman Alhajahjeh, Majedah Hmeidan, Mus'ab Elatrsh, Faris Al-Abbadi, Diala Kakish, Razan Sukerji, Mohammad Salah, Bashir Al Hussein Al Awamlh, David I. Lee, Mohammed Shahait

**Affiliations:** ^1^ Center for Anesthesia Research Excellence (CARE), Beth Israel Deaconess Medical Center, Harvard Medical School, Boston, MA; ^2^ Department of Internal Medicine, King Hussein Cancer Center (KHCC), Amman, Jordan; ^3^ School of Medicine, University of Jordan, Amman, Jordan; ^4^ Jordan Red Crescent Hospital, Amman, Jordan; ^5^ School of Medicine, University of Debrecen, Debrecen, Hungary; ^6^ Kindi Hospital, Manama, Bahrain; ^7^ Al Bashir Hospital, Internship, Amman, Jordan; ^8^ Department of Urology, Vanderbilt University Medical Center, Nashville, TN; ^9^ Department of Urology, University of California, Irvine, CA; ^10^ Department of Urology, Clemenceau Medical Center, Dubai, UAE; ^11^ School of Medicine, University of Sharjah, Sharjah, UAE

## Abstract

**PURPOSE:**

Clinical trials are valuable evidence for managing urologic malignancies. Early termination of clinical trials is associated with a waste of resources and may substantially affect patient care. We sought to study the termination rate of urologic cancer clinical trials and identify factors associated with trial termination.

**METHODS:**

A cross-sectional search of ClinicalTrials.gov identified completed and terminated kidney, prostate, and bladder cancer clinical trials started. Trials were assessed for reasons for termination. Multivariable analyses were conducted to determine the significant factors associated with the termination.

**RESULTS:**

Between 2000 and 2020, 9,145 oncology clinical trials were conducted, of which 11.30% (n = 1,033) were urologic cancer clinical trials. Of the urologic cancer clinical trials, 25.38% (n = 265) were terminated, with low patient accrual being the most common reason for termination, 52.9% (n = 127). Multivariable analysis showed that only the university funding source odds ratio (OR) of 2.20 (95% CI, 1.45 to 3.32), single-center studies OR of 2.11 (95% CI, 1.59 to 2.81), and sample size of <50 were significant predictors of clinical trial termination OR of 5.26 (95% CI, 3.85 to 7.69); all *P* values are <.001.

**CONCLUSION:**

The termination rate of urologic cancer clinical trials was 25%, with low accrual being the most frequently reported reason. Trials funded by a university, single-center trials, and small trials (sample size <50) were associated with early termination. A better understanding of these factors might help researchers, funding agencies, and other stakeholders prioritize resource allocations for multicenter trials that aim to recruit a sufficient number of patients.

## Teaser

Urological cancer trial termination at 25% due to low accrual. Factors: university funding, single-center, small sample.

## INTRODUCTION

In the past 30 years, there has been a global trend toward increasing incidence of kidney, bladder, and prostate cancers, collectively representing the most common genitourinary cancers.^1^ It was estimated that the incidence was 145,910, 234,750, and 524,110 for kidney, bladder, and prostate cancer cases, respectively, between 1990 and 2019.^2^ Consequentially, the socioeconomic burden of treatment for genitourinary (GU) malignancies is soaring.^3^ For example, bladder cancer's economic burden is considered the highest per patient of all cancers, with almost 8.6 billion euros per year in the United States and Europe.^4,5^

**CONTEXT**

**Key Objective**
What are the factors associated with the termination rate of urologic cancer clinical trials?
**Knowledge Generated**
The overall termination rate of urologic cancer clinical trials was 25.4%. The most frequent causes for trial termination were low patient accrual and safety concerns. Trials that received university funds, were based in a single center, and aimed to recruit <50 patients were more likely to end up being terminated.
**Relevance**
A better understanding of these factors might help researchers, funding agencies, and other stakeholders prioritize resource allocations for multicenter trials that aim to recruit a large number of patients.

Randomized clinical trials remain the cornerstone in advancing cancer care as they provide high-level evidence that influences the management of thousands of patients globally.^6^ For example, landmark clinical trials investigating the use of novel hormonal therapy in advanced prostate cancer led to a significant increase in overall survival rates for patients.^7^ Nevertheless, randomized control trials (RCTs) require a robust institutional infrastructure, support personnel, funding, and time and effort from the recruiting physician.^6,8^ Moreover, they may result in psychological, physical, and financial burdens for participants.^9^

Clinical trial termination because of safety issues, financial strains, and other logistical factors is well studied. Around 22% of oncologic clinical trials experience termination, whereas for nononcology clinical trials, the termination rate is approximately 19%.^10^ While clinical trial termination might be associated with a sense of loss, anxiety, and disappointment for patients, it also has an economic impact associated with the cost, utilization of research infrastructure, and loss of opportunity to extend life or improve life quality. However, most studies that assessed clinical trial termination were not focused on GU cancer clinical trials.

In this context, we sought to study the termination rate of GU cancer clinical trials, specifically among kidney, bladder, and prostate cancers. The study aimed to identify factors associated with clinical trial termination that can be improved for future trial design.

## METHODS

### Data Source and Search Study

ClinicalTrials.gov is the most comprehensive clinical trial register that provides extensive information to the public.^11^ The investigators independently conducted a thorough review of clinical trials of kidney, bladder, and prostate cancers available on the registry. The investigators initiated the search on March 11, 2023, focusing on identifying completed trials and having their results posted before December 2020. Only trials that had a recruitment status identified as “completed”; “active, not recruiting”; “terminated”; “suspended”; and “Withdrawn” on ClinicalTrials.gov have been included.

### Data Extraction

#### Data Related to Clinical Trial

The extracted data included several parameters such as clinical trial date, funding source, intervention, enrollment, sample size, trial completion date, phase, clinical trial status, the reason for discontinuation, masking, center(s) (single or multicenter), area of the clinical trial, and primary outcome of the trial. Phases I/II and II/III were defined as phases II and III, respectively. The funding source was categorized into National Institutes of Health (NIH), non-NIH US funding or other (non-NIH, non-US) funding, industrial companies, private institutions/hospitals, or universities. The intervention was classified into medical, surgical, diagnostic, palliative, and other. Moreover, the area of conducting the clinical trials was categorized on the basis of high-income countries (HICs) or low- and middle-income countries (LMICs), according to the 2022-2023 World Bank Atlas country's income-level classification.

#### Data Related to the Principal Investigator

We used the Wiki-Gendersort to check the sex of the principal investigator (PI), either female or male,^12^ the age of the PI, the total number of citations, and the level of experience of the PI, which has been evaluated by subtracting the year of graduation of the PI from medical school or any similar-level degree.

Two independent investigators conducted the data extraction, and a third investigator was consulted to resolve any disagreements. In the case of clinical trial termination, the reason was extracted as provided on ClinicalTrials.gov. Reasons for termination included financial constraints, administrative reasons, informative decisions, low accrual, patients' safety, and other/unclear reasons.

### Ethical Statement

As a result of the nature of the study, no IRB approval was needed to conduct this scientific work.

### Consent for Publication

The data that have been collected from the clinical trials are published and publicly available, and thus, no informed consent was needed.

### Statistical Analysis

The study categorized trials as completed or discontinued and reported continuous variables as median with IQR and categorical variables as percentages (%). Chi-square tests were used to determine differences between categorical variables, whereas the Shapiro-Wilks test assessed normal distribution for continuous variables. If variables were not normally distributed, logarithmic conversion was applied to adjust for normal distribution. Independent *T*-tests were used to compare two groups, and one-way ANOVA was used for more than two groups. A multivariable logistic regression analysis was conducted to identify the most factors associated with clinical trial termination, including centers (single *v* multiple), funding source, type of intervention, number of agents, masking of the clinical trial, number of participants, and the phase of the trial. R software was used for all statistical analyses, with *P* values <.05 considered statistically significant.

## RESULTS

**FIG 1:**
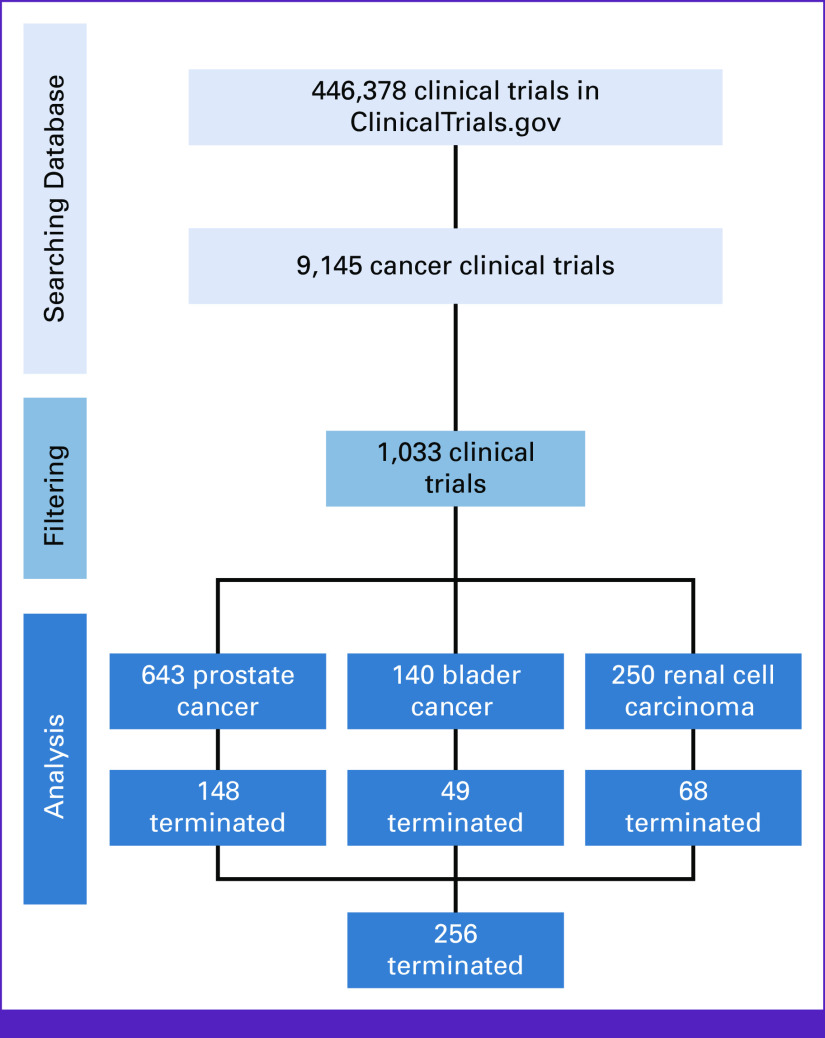
Flowchart of included clinical trials collection. This figure presents a flowchart depicting the systematic process for the collection of included clinical trials in a comprehensive review. The flowchart comprises a series of sequential steps, each of which contributes to the selection of relevant clinical trials for analysis and synthesis.

Between 2000 and 2020, 9,145 oncology clinical trials were conducted, of which 11.30% (n = 1,033) focused on urologic cancers. Prostate cancer clinical trials constituted the majority, with 62.24% (n = 643), followed by renal cell carcinoma clinical trials with 24.20% (n = 250) and bladder cancer clinical trials with 13.41% (n = 140; Fig 1). These clinical trials recruited 5,093,717 patients, with a median sample size of 41 and IQR 17-107, and more than half, 55.5% (n = 573), recruited <50 patients. Single-arm interventions were conducted in 45.7% (n = 402) of the trials, and medical intervention was the most common type of intervention in 79.5% (n = 816), with most of them being phase II clinical trials in 76.2% (n = 645). Industrial companies were the most common funding source, 51.2% (n = 528), followed by NIH 27.8% (n = 287). Throughout the trial conduction, most trials had no masking, 83.6% (n = 851), and more than half were conducted in more than one center, 55.5% (n = 578). Only 2.27% (n = 20) of clinical trials were conducted in LMICs. Table 1 summarizes the characteristics of the urologic clinical trials included in the study.

**TABLE 1:** Full Characteristics of the Clinical Trials

Characteristic	All Trials (n = 1,033), No. (%)
Sex of the PI	
Female	137 (16.8)
Male	679 (83.2)
Experience in the medical field	20.2 (9.71)
PI age, years	59.0 (52.0; 64.5)
PI number of citations	7,407 (2,525; 17,985)
Funding source	
Industrial	528 (51.2)
NIH	287 (27.8)
Other	2 (0.19)
Private	90 (8.73)
University	124 (12.0)
Intervention	
Device	31 (3.02)
Medication	816 (79.5)
Medication/surgery	41 (3.99)
Medication/radiation	25 (2.43)
Other	57 (5.55)
Radiation	34 (3.31)
Surgery	23 (2.24)
No. of agents	
1	402 (45.7)
2	298 (33.9)
3	113 (12.9)
More than 3	66 (7.51)
Phase	
I	45 (5.31)
II	645 (76.2)
III	135 (15.9)
IV	22 (2.60)
Reason for discontinuation	
Administrative reasons	26 (10.8)
Funding	27 (11.2)
Low accrual	127 (52.9)
Other	12 (5.00)
Patient safety	48 (20.0)
Masking	
None	851 (83.6)
Single	26 (2.55)
Double	55 (5.40)
Triple	41 (4.03)
Quadruple	45 (4.42)
Centers	
Multi	558 (55.5)
Single	448 (44.5)
Country	
HICs	860 (97.7)
LMICs	20 (2.27)
Sample	
Less than 50	573 (55.5)
More than 50	460 (44.5)

Abbreviations: HICs, high-income countries; LMICs, low- and middle-income countries; NIH, National Institutes of Health; PI, principal investigator.

Of all the urologic cancer clinical trials, 25.38% (n = 265) were terminated; low patient accrual was the most common reason, 52.9% (n = 127), followed by patient safety concerns, 20.0% (n = 48; Fig 1). A total of 12,280 patients were enrolled in these trials (Tables 2 and 3). Univariable analysis revealed significant associations between university funding of the clinical trials (18.9% *v* 9.66%; *P* < .001, for terminated *v* completed), surgical intervention (3.02% *v* 1.97%; *P* = .020, for terminated *v* completed), a higher number of arms with more than three interventions (9.40% *v* 6.82%; *P* < .001, for terminated *v* completed), phase II trials (84.8% *v* 73.0%; *P* = .005, for terminated vs completed), absence of masking (90.1% *v* 81.3%; *P* = .014, for terminated *v* completed), single-center clinical trials (58.2% *v* 39.7%; *P* < .001, for terminated *v* completed), and clinical trials recruiting less than 50 patients (82.3% *v* 46.2%; *P* < .001, for terminated vs completed). More details are shown in Table 1. On multivariable analysis, university-based funded trials (odds ratio [OR], 2.20 [95% CI, 1.45 to 3.32]; *P* < .001), single-center studies (OR, 2.11 [95% CI, 1.59 to 2.81]; *P* < .001), and trials with a sample size <50 (OR, 5.26 [95% CI, 3.85 to 7.69]; *P* < .001) were associated with termination (Table 1).

**TABLE 2:** Univariate Analysis for the Difference Between Completed and Terminated Clinical Trials

Factor	Completed Trial (n = 768)	Terminated Trial (n = 265)	*P*
Sex of the PI, No. (%)			.585
Female	102 (17.3)	35 (15.4)	
Male	487 (82.7)	192 (84.6)	
Experience in the medical field, No. (%)	19.6 (9.57)	21.5 (9.94)	.056
PI age, years	59.0 (52.0; 65.0)	59.0 (53.0; 64.0)	.640
PI number of citations	7,687 (2,865; 17,985)	6,230 (2,101; 18,157)	<.001*
Funding source, No. (%)			
Industrial	404 (52.7)	124 (46.8)	
NIH	212 (27.7)	75 (28.3)	
Other	1 (0.13)	1 (0.38)	
Private	75 (9.79)	15 (5.66)	
University	74 (9.66)	50 (18.9)	
Intervention, No. (%)			.020*
Device	24 (3.15)	7 (2.64)	
Medication	602 (79.0)	214 (80.8)	
Medication/surgery	26 (3.41)	15 (5.66)	
Medication/radiation	19 (2.49)	6 (2.26)	
Other	53 (6.96)	4 (1.51)	
Radiation	23 (3.02)	11 (4.15)	
Surgery	15 (1.97)	8 (3.02)	
No. of agents, No. (%)			<.001*
1	300 (46.5)	102 (43.6)	
2	218 (33.8)	80 (34.2)	
3	83 (12.9)	30 (12.8)	
More than 3	44 (6.82)	22 (9.40)	
Phase, No. (%)			.005*
I	36 (5.78)	9 (4.02)	
II	455 (73.0)	190 (84.8)	
III	113 (18.1)	22 (9.82)	
IV	19 (3.05)	3 (1.34)	
Masking, No. (%)			.014*
None	615 (81.3)	236 (90.1)	
Single	23 (3.04)	3 (1.15)	
Double	44 (5.82)	11 (4.20)	
Triple	37 (4.89)	4 (1.53)	
Quadruple	37 (4.89)	8 (3.05)	
Centers, No. (%)			<.001*
Multi	448 (60.3)	110 (41.8)	
Single	295 (39.7)	153 (58.2)	
Country, No. (%)			.009*
HICs	610 (96.8)	250 (100)	
LMICs	20 (3.17)	0 (0.00)	
Sample, No. (%)			<.001*
Less than 50	355 (46.2)	218 (82.3)	
More than 50	413 (53.8)	47 (17.7)	

Abbreviations: HICs, high-income countries; LMICs, low- and middle-income countries; NIH, National Institutes of Health; PI, principal investigator.

* Signifies statistical significance.

**TABLE 3:** Multivariate Analysis for the Difference Between Completed and Terminated Clinical Trials

Multivariate Analysis	OR (95% CI)	*P*
Funding source		
Industrial	Ref	Ref
NIH	1.15 (0.83 to 1.60)	.402
Other	3.25 (0.08 to 127)	.472
Private	0.66 (0.35 to 1.16)	.150
University	2.20 (1.45 to 3.32)	<.001*
Intervention		
Device	Ref	Ref
Medication	1.20 (0.53 to 3.09)	.675
Medication/surgery	1.94 (0.69 to 5.95)	.215
Medication/radiation	1.08 (0.29 to 3.89)	.901
Other	0.27 (0.06 to 0.99)	.049
Radiation	1.62 (0.53 to 5.18)	.398
Surgery	1.80 (0.53 to 6.30)	.344
No. of agents		
1	Ref	Ref
2	1.08 (0.77 to 1.52)	.661
3	1.07 (0.65 to 1.70)	.794
More than 3	1.47 (0.83 to 2.56)	.183
Phase		
I	Ref	Ref
II	1.65 (0.81 to 3.73)	.176
III	0.77 (0.33 to 1.93)	.568
IV	0.65 (0.13 to 2.56)	.557
Masking		
None	1.52 (0.80 to 3.15)	.213
Single	0.54 (0.11 to 1.98)	.373
Double	Ref	Ref
Triple	0.45 (0.11 to 1.45)	.185
Quadruple	0.87 (0.30 to 2.41)	.789
Centers		
Multi	Ref	Ref
Single	2.11 (1.59 to 2.81)	<.001*
Sample		
Less than 50	Ref	Ref
More than 50	0.19 (0.13 to 0.26)	<.001*

Abbreviations: NIH, National Institutes of Health; OR, odds ratio.

* Signifies statistical significance.

## DISCUSSION

To our knowledge, this is the first comprehensive study that examined the factors associated with GU cancer clinical trial termination. The overall termination rate of these trials was 25.4%. The most frequent causes for trial termination in this study were low patient accrual and safety concerns. Trials that receive university funds, were based in a single center, and aimed to recruit <50 patients were more likely to end up being terminated.

We observed that almost a quarter of clinical trials accounting for urologic malignancies conducted between 2000 and 2020 were terminated, which is consistent with the worldwide reported termination rate of nonurology oncologic clinical trials.^13^ This finding emphasizes the sophistication encountered in the designing and successfully conducting this specialized subset of clinical trials, given the complexity of urologic cancers. We observed that the predominant reason leading to the termination of urologic malignancies' clinical trials was low patient accrual (53%), followed by patient safety concerns (20%) of the trials. These findings are distinctly different from the predominant reasons leading to the termination of nononcologic trials, where the main reason for termination is the lack of efficacy.^14-17^ This could be of particular importance given that malignancies, including urologic malignancies, progress at a widely variable and, in certain instances, unexpected rate and high patient accrual is mandated to achieve high evidence-based and accurate findings.^18-20^ In addition, safety concerns are paramount, especially in malignancies, as patients tend to be immunocompromised and frail, making them prone to rapid deconditioning. Therefore, tailored protocols with rigorous safety measures must be implemented to ensure the safety of enrolled participants.^21^

We observed the presence of several factors associated with the termination of GU clinical trials across the conducted trials between 2000 and 2020. The funding source was found to have a notable connection with the increased probability of clinical trial termination. As such, in university-funded trials, 18.9% (n = 50) were terminated, while only 9.66% (n = 74) were completed. However, it is noteworthy that there are considerable discrepancies and a lack of consensus regarding the impact of the funding source and the outcome of the trials in terms of successful completion or termination and the currently available literature does not completely explain the wide variation witnessed.^14,15,22,23^ Furthermore, our study revealed that the setting of the clinical trial and whether it is a multicenter trial or a single-center trial are significantly associated with the outcome of the trial as only 19.7% of the multicenter clinical trials were terminated in contrast to 31.4% of the single-center clinical trials. Indeed, our finding of a lower termination rate in multicenter clinical trials when compared with a single center is in line with previously reported literature.^24-27^ While multicenter clinical trials are challenging, require robust infrastructure, and necessitate intricate logistics in contrast to single-center trials, the lower termination among multicenter trials suggests that single centers may collaborate with others to improve study completion.

Notably, a staggeringly low number of clinical trials were conducted in LMICs, accounting for only 2.27% of all the reported trials during 2000-2020. When compared with clinical trials conducted in HICs, our study demonstrated that there is a statistically significant association between the likelihood of completion of the clinical trial and whether it is conducted in an LMIC or HIC as surprisingly, none of the trials conducted in LMICs were terminated, in contrast to 250 trials (29%) of those conducted in HICs were terminated. Nonetheless, this is postulated to be due to a multitude of factors including the small number of clinical trials conducted in LMICs in contrast to HICs and thus the potential lack of generalizability; in addition to that, further emphasis on stringent and vigorous protocols might have been placed in the studies conducted in the LMICs given the scarcity of resources and thus the desirability to complete the trials.

This study is limited by a multitude of factors, notably the restricted time of 2000-2020 and thus the exclusion of clinical trials conducted before and after the allocated period, in addition to the exclusion of non-English reported trials. Furthermore, it is noteworthy that this work focused on establishing the predominant factors behind the termination of the clinical trials on the basis of the publicly available data and did not count for external factors that could potentially be contributing factors to the termination of trials. Nonetheless, the presented results are of significant and paramount importance toward improving successful conduction of GU cancer clinical trials as evidenced by their consistency in certain aspects with previously conducted studies evaluating the termination of nonurologic condition clinical trials, including oncologic trials.^14,15,28^ In addition, the absence of studies evaluating the driving factors leading to the termination of urologic malignancies' clinical trials further augments the significance of the findings reported in this work because of the importance of clinical trials in the progression and advancement of care in urologic malignancies. Accordingly, the reported findings can be of significant value in designing and conducting future clinical trials targeting urologic malignancies.

In conclusion, the termination rate of urologic cancer clinical trials was 25%, with low accrual being the most frequently reported reason. Trials funded by a university, single-center trials, and small trials (sample size <50) were associated with early termination. A better understanding of these factors might help researchers, funding agencies, and other stakeholders to prioritize resource allocations for multicenter trials that aim to recruit a large number of patients.

## Data Availability

All data generated or analyzed during this study are fully included in this published article. Researchers or interested parties who are seeking access to the raw data sets used and/or analyzed during the current study can obtain them from the corresponding author upon a reasonable request.
